# Measurement properties of the Brazilian versions of Fear-Avoidance Beliefs Questionnaire and Tampa Scale of Kinesiophobia in individuals with shoulder pain

**DOI:** 10.1371/journal.pone.0260452

**Published:** 2021-12-01

**Authors:** Danilo Harudy Kamonseki, Melina Nevoeiro Haik, Larissa Pechincha Ribeiro, Rafaela Firmino de Almeida, Lucas Araújo de Almeida, Carlos Luques Fonseca, Paula Rezende Camargo

**Affiliations:** 1 Laboratory of Analysis and Intervention of the Shoulder Complex, Universidade Federal de São Carlos (UFSCar), São Carlos, (SP), Brazil; 2 Faculdade Anhanguera, Sorocaba, São Paulo, Brazil; Universiti Sains Malaysia, MALAYSIA

## Abstract

**Purpose:**

To verify the measurement properties of the Brazilian versions of Fear-avoidance Beliefs Questionnaire (FABQ) and Tampa Scale of Kinesiophobia (TSK) in individuals with shoulder pain.

**Methods:**

Individuals with shoulder pain (>18 years) were included in this study. Structural validity was verified by exploratory factor analysis, which was used to identify dimensionality of the FABQ and TSK. Test-retest reliability was assessed with intraclass correlation coefficient_(3,1)_ and internal consistency with Cronbach’s alpha. Floor or ceiling effects were also investigated. Responsiveness was verified by effect sizes and area under the receiver operating characteristic curve (AUC).

**Results:**

Exploratory factor analysis identified two and one factor in the FABQ and TSK, respectively. FABQ and TSK presented moderate to good reliability and adequate internal consistency (Cronbach’s alpha > 0.70). The floor effect was present in one factor of the FABQ. The FABQ and TSK showed small to moderate effect sizes and did not show adequate AUC.

**Conclusion:**

FABQ and TSK are multidimensional and unidimensional instruments, respectively. Those instruments presented moderate to good reliability and the responsiveness was considered to be suboptimal in individuals with shoulder pain.

## Introduction

Shoulder pain is a common complaint [1,2] that may have a negative impact on sports performance, work productivity [3,4], functional activities [5], and healthcare expenses over time [6]. Psychological factors have been associated with pain intensity and disability in individuals with shoulder pain [7]. The fear of pain or potential threats may induce catastrophic thoughts, which is associated with the development of avoidance behaviors [8,9]. Individuals with catastrophic thoughts tend to have hypervigilance behavior to avoid worsening of the symptoms and may withdraw from social activities [10], which may contribute to physical inactivity, depression, and disability [10]. Fear-avoidance beliefs may be related to physical or work activities that may elicit or worse symptoms, and kinesiophobia has been defined as the extreme fear of movement due to the sense of vulnerability to an injury/reinjury [8,9]. Assessment of fear-avoidance and kinesiophobia is of great importance in the clinical practice to support the rehabilitation process when those psychological impairments are present [10,11].

The Fear-Avoidance Beliefs Questionnaire (FABQ) is a self-reported questionnaire that was initially developed to assess the fear-avoidance beliefs related to physical activity and work in individuals with low back pain [12]. The Tampa Scale of Kinesiophobia (TSK) is a patient-reported outcome measure developed to assess kinesiophobia in individuals with chronic pain [13]. Both questionnaires have been translated and validated into several languages [14–21]. The literature recommends that the psychometric properties should be tested when an outcome measure is used in a different population or language [22,23]. The psychometric properties of those questionnaires were verified in individuals with different disorders, such as low back pain [16,21,24,25], neck pain [26,27], fibromyalgia syndrome [28], and temporomandibular disorders [14,29]. Although the FABQ and TSK were already translated into Brazilian Portuguese [21,29–31], their psychometric properties have not been established in individuals with shoulder pain yet. Therefore, the purpose of this study was to verify the structural validity, reliability, and responsiveness of the Brazilian versions of FABQ and TSK in individuals with shoulder pain.

## Methods

This is a study on measurement properties that included cross-sectional and longitudinal study design. This is a secondary analysis of previous studies [32–34] performed at the Laboratory of Analysis and Intervention of the Universidade Federal de São Carlos (UFSCar). All original studies and the current one were approved by the Human Research Ethics Committee of the University (protocol number: CAAE 21958819.5.0000.5504). The individuals included in the original studies were recruited through flyers placed at the University buildings, local orthopedic clinics, and advertisements in local newspapers and radio, and online resources (eg, university intranet and social media). All individuals signed a written consent before study enrollment and were aware of the main purpose of the study. Participants had to be older than 18 years, report shoulder pain during arm elevation of at least 3 points measured by 11-point Numerical Pain Rating Scale (NPRS), and be seeking for physical therapy to treat their shoulder pain. Individuals were excluded if they presented a history of clavicle, scapula or humerus fracture, and/or surgery in the shoulder region, shoulder dislocation or instability based on positive apprehension test and/or sulcus test, and massive rotator tears based on a positive drop-arm test and/or shoulder pseudoparalysis, pregnancy, frozen shoulder, numbness or tingling of the upper limb reproduced by the cervical compression test or upper limb tension test, diabetes, rheumatologic or neurologic illness, and performed physical therapy within 6 months prior to the study [34].

The analysis using classical test theory of a patient report outcome measure is suggested to be performed in at least 50 individuals [35]. Moreover, when using a factorial analysis, the recommended sample size is 150 individuals or more, and 10 individuals should be included per item being analyzed [36]. The total sample was composed by 178 individuals that were analyzed for structural validity. Of those 178 individuals, 86 agreed to participate in the reliability, and 59 in the responsiveness analysis.

### Outcome measures

#### Fear-Avoidance Beliefs Questionnaire

The Fear-Avoidance Beliefs Questionnaire (FABQ) is a self-reported questionnaire with 16 items [30]. Higher scores of this questionnaire indicate worse fear-avoidance beliefs. The items of the FABQ with word “back” with were modified and replaced by “shoulder”. The test-retest reliability of the Brazilian version of the FABQ was tested in two studies with individuals with low back pain [21,30], and showed an intraclass correlation coefficient (ICC) of 0.84 [30] and 0.94 [21] for physical activity subscale, and 0.91 [30] and 0.82 [21] for work subscale.

#### Tampa Scale for Kinesiophobia

The Tampa Scale for Kinesiophobia (TSK) is a self-reported instrument, and the original version was composed of 17 questions. However, past studies [37,38] showed that TSK has better structural validity when some items were excluded. Therefore, TSK with 11 items (TSK-11) was used in this study, which excludes items 4, 8, 9, 12, 14, and 16 from the original 17-item instrument [37,39]. However, the versions of TSK with 17, 13, and 12 items were also used in the confirmatory factor analysis to verify which version presents a better model fit. The score is obtained by summing all questions, and higher scores indicate worse kinesiophobia [21]. The test-retest reliability of the Brazilian version of the TSK was tested in individuals with fibromyalgia and showed an ICC of 0.85 [31].

#### Pain intensity

The most frequent pain intensity of the shoulder during the past week was assessed using the NPRS of 11 points. The NPRS score ranges from 0 to 10, with higher scores indicating worse symptoms. This scale is a valid and reliable scale for individuals with shoulder pain, with a test-retest that showed an ICC of 0.84 [40].

#### Function of the upper limbs

Function of the upper limbs was assessed with the Brazilian version of Disabilities of the Arm, Shoulder, and Hand (DASH). The DASH is a self-reported questionnaire with 30 items. The score ranges from 0 to 100 and higher scores indicate worse function. The reliability of the Brazilian DASH questionnaire showed an ICC of 0.90 [41].

#### Quality of life

The quality of life was assessed with the Brazilian version of EuroQol-5D-3L, which is a questionnaire with five dimensions: mobility, self-care, usual activities, pain/discomfort, and anxiety/depression [42]. The five domains are combined to represent the health status with a score that ranges from 0 to 1 and higher scores indicate better quality of life [42]. The test-retest reliability of the Brazilian version of EuroQol-5D-3L showed an ICC of 0.85 [43].

#### Global Rating of Change Scale

The Global Rating of Change Scale (GROC) measures the individual’s perception of improvement or worsening over time (from baseline to 8 weeks after treatment). This scale ranges from -7 to 7, with positive and higher scores indicating a perception of improvement, negative and lower scores indicating perception of worsening, and zero indicates no change [44].

### Statistical analysis

Mean and standard deviation (SD) were calculated for continuous data. Data normality was verified by the Shapiro-Wilk test. The FABQ, TSK, NPRS, DASH, and EuroQol-5D-3L presented scores with non-normal distribution (p > 0.05) and were analyzed using non-parametric tests. The exploratory factor analysis was performed by using the software FACTOR (Universitat Rovira i Virgili, Tarragona, Spain). The Statistical Package for the Social Sciences (SPSS Inc, Chicago, IL) version 23 was used for the other analyses. The level of significance was set at 0.05 for all statistical analyses.

#### Structural validity

The structural validity of FABQ and TSK was assessed by the exploratory factor analysis. The exploratory factor analysis established which items of the questionnaire were contributing to a factor [36]. To be considered suitable for the exploratory factor analysis the data should attend the following criteria: correlation matrix showing coefficients greater than 0.4, significant Bartlett’s test of sphericity (p < 0.05), and Kaiser-Meyer-Olkin test greater than 0.6 [45,46]. The exploratory factor analysis was performed with polychoric matrix, robust diagonally weighted least squares extraction method and robust promin rotation. The number of factors was determined using Catell’s scree test and the eigenvalue value was determined using parallel analysis. The items with factor loading greater than 0.3 were included in the factor and the items with communality score < 0.40 were deleted [47].

#### Reliability

The FABQ and TSK were applied twice by the same examiner under similar conditions (self-administered in a laboratory setting), with a mean interval of 7.55 ± 3.34 days between applications. The individuals did not receive treatment for shoulder pain during this period. The time between applications prevented recall and changes in clinical condition [35].

Test-retest reliability was evaluated using ICC_(3,1)_ with values interpreted as follows: less than 0.50 as poor, 0.50 to 0.75 as moderate, 0.75 to 0.90 as good, and 0.90 as excellent reliability [48]. The internal consistency was evaluated using Cronbach’s alpha, and it was considered adequate when greater than 0.70 [35]. The standard error of the measurement (SEM) was calculated with the formula SEM = SD √1 –ICC. Minimal detectable change (MDC) was also calculated for FABQ and TSK using the formula MDC_90_ = SEM x √2 x 1.64 [49,50].

#### Interpretability

The ceiling and floor effects were considered present if more than 15% of the total sample achieved the lowest or highest possible total score [35].

#### Responsiveness

The individuals who participated in the responsiveness phase received eight weeks of treatment based on exercises. Responsiveness was analyzed using the effect size (ES), standardized response mean (SRM), and the area under the curve (AUC) of the receiver operating characteristic (ROC). ES and SRM were interpreted as follows: less than 0.50 as small, 0.50 to 0.80 as moderate, and greater than 0.80 as large responsiveness [35,49–51].

A ROC curve was plotted based on the external anchor (GROC). The external anchor classified the individuals into two categories: “importantly improved” or “slightly improved or not improved”, considering a cut-off of 4 points [52,53]. The AUC and 95% confidence interval were calculated using the change scores, and AUC greater than 0.70 was considered as adequate responsiveness [35,49–51].

## Results

The characteristics of the individuals are presented in Table 1.

**Table 1:** Characteristics of the individuals according to the analyzed measurement property.

Characteristics	Structural Validity (n = 178)	Reliability (n = 86)	Responsiveness (n = 59)
Age, years	39.70 ± 14.01	40.68 ± 14.27	37.71 ± 12.5
Sex, female (%)	74 (41.60)	43 (50.00)	32 (54.23)
Body mass index, Kg/m^2^	25.52 ± 3.55	25.58 ± 3.39	24.72 ± 2.49
Duration of symptoms, months	38.15 ± 51.78	28.07 ± 37.19	25.86 ± 36.20
Most painful side, n (%)			
Dominant	115 (64.60)	57 (66.27)	33 (55.93)
Non-dominant	63 (35.4)	29 (33.72)	26 (44.06)
Symptoms, n (%)			
Bilateral	42 (23.70)	20 (23.25)	13 (22.03)
Unilateral	136 (76.4)	66 (76.74)	46 (77.96)
Educational level, n (%)			
Incomplete elementary	7 (3.90)	2 (2.32)	1 (1.69)
Elementary	14 (7.90)	8 (9.30)	1 (1.69)
High school	72 (40.4)	34 (39.53)	23 (38.98)
University degree	85 (47.80)	42 (48.83)	34 (57.62)
Numerical Pain Rating Scale, (0–10)	5.15 ± 2.45	5.62 ± 2.60	5.25 ± 2.41
DASH questionnaire, (0–100)	30.36 ± 19.41	35.88 ± 18.82	35.57 ± 15.34
EuroQol-5D-3L, (0–1)	0.76 ± 0.16	0.73 ± 0.17	0.75 ± 0.13

Continuous data are reported as mean ± standard deviation. Categorical variables are presented as count and percentage. Abbreviations: DASH, Disabilities of the Arm, Shoulder, and Hand. Higher scores of Numerical Pain Rating Scale and DASH questionnaire indicate worst condition. Higher scores of EuroQol-5D-3L indicate better condition.

**Fig 1:**
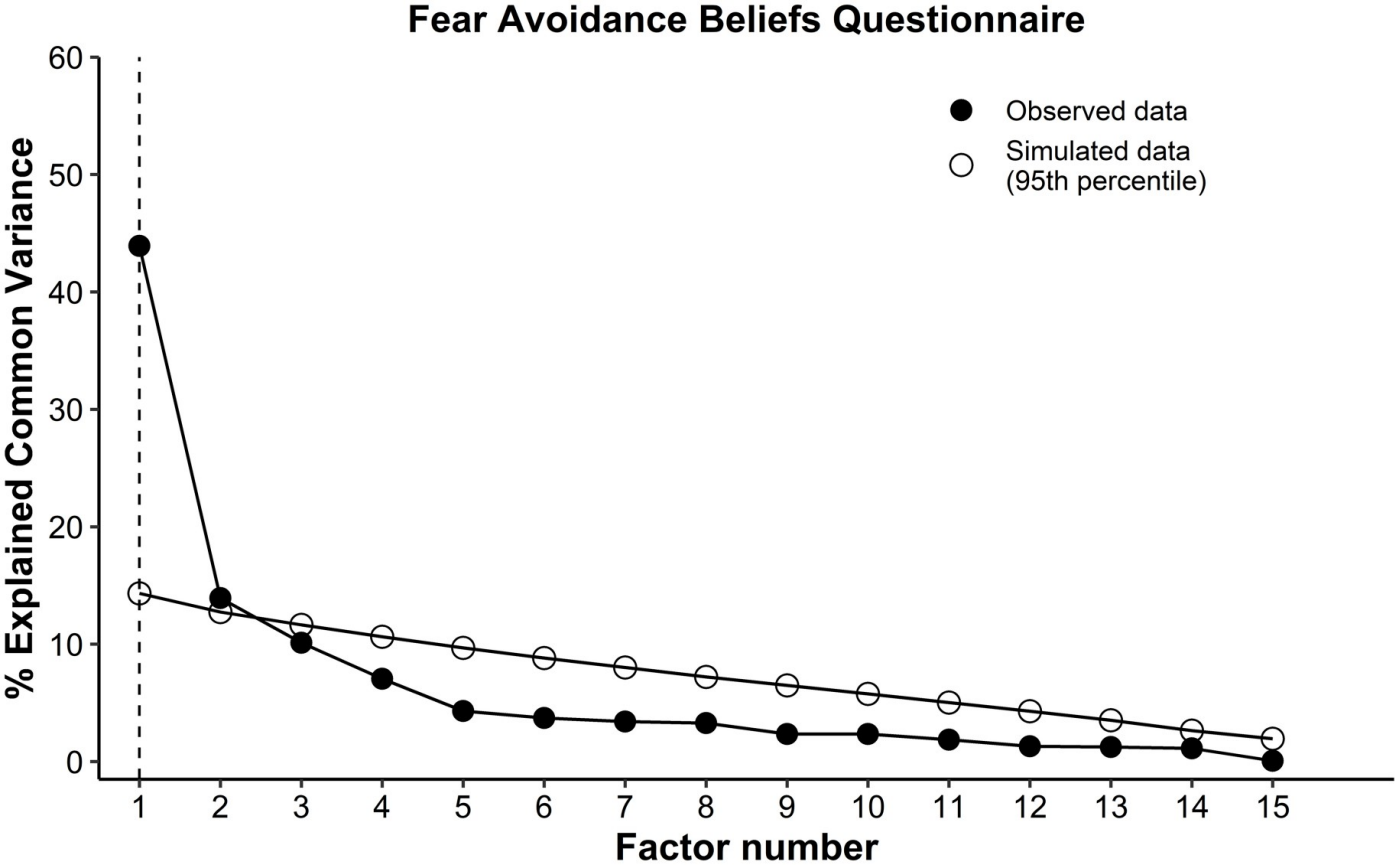
Parallel analysis of the Fear Avoidance Beliefs-Questionnaire indicated two-factors structure. The factors above the simulated data (95^th^ percentile) indicated the number of the factors in the structure.

The FABQ and TSK were considered suitable for the exploratory factor analysis. The FABQ showed statistical significance on the Bartlett’s sphericity test (p = 0.0001), and the Kaiser-Meyer-Olkin test was 0.86. The exploratory factor analysis suggested 2 factors (Fig 1) that accounted for 56.4% of total variance. Factor 1 was comprised of 5 items (items 1 to 5) and accounted 43% of the variance, factor 2 was comprised of 11 items (items 6 to 16) and accounted 13% of the variance (Table 2). The items 1, 2, 3, 8, 15, and 16 presented communalities lower than 0.4. Therefore, a new model (model 2) was proposed excluding those items with low value of communality and reanalyzed with an additional exploratory factor analysis, which indicated that the two factors accounted 72% of the variance. This new model with 2 factors (factor 1 considered the items 6, 7, 9, 10, 11, 12, 13, and 14, and the factor 2 considered the items 4 and 5) was considered in the analysis of the reliability, internal consistency, and responsiveness.

**Table 2:** Exploratory factor analysis of Fear-Avoidance Beliefs Questionnaire (n = 178).

Items	Factor loading		Communality
	Factor 1 (FABQ work)	Factor 2 (FABQ Physical activity)	
Item 1	**-0.44**	**0.45**	0.26
Item 2	-0.20	**0.58**	0.28
Item 3	-0.08	**0.64**	0.38
Item 4	0.05	**0.66**	0.46
Item 5	0.22	**0.61**	0.52
Item 6	**0.80**	-0.33	0.55
Item 7	**0.89**	-0.31	0.68
Item 8	**0.46**	-0.02	0.21
Item 9	**0.75**	0.01	0.57
Item 10	**0.84**	-0.11	0.65
Item 11	**0.81**	0.00	0.66
Item 12	**0.87**	0.10	0.83
Item 13	**0.86**	0.11	0.81
Item 14	**0.78**	0.14	0.71
Item 15	**0.55**	0.12	0.37
Item 16	**0.50**	0.05	0.28
Eigenvalue	6.47	1.56	
Variance, %	43.92	13.93	

Note: Factor loadings in bold indicate values above 0.4.

**Fig 2:**
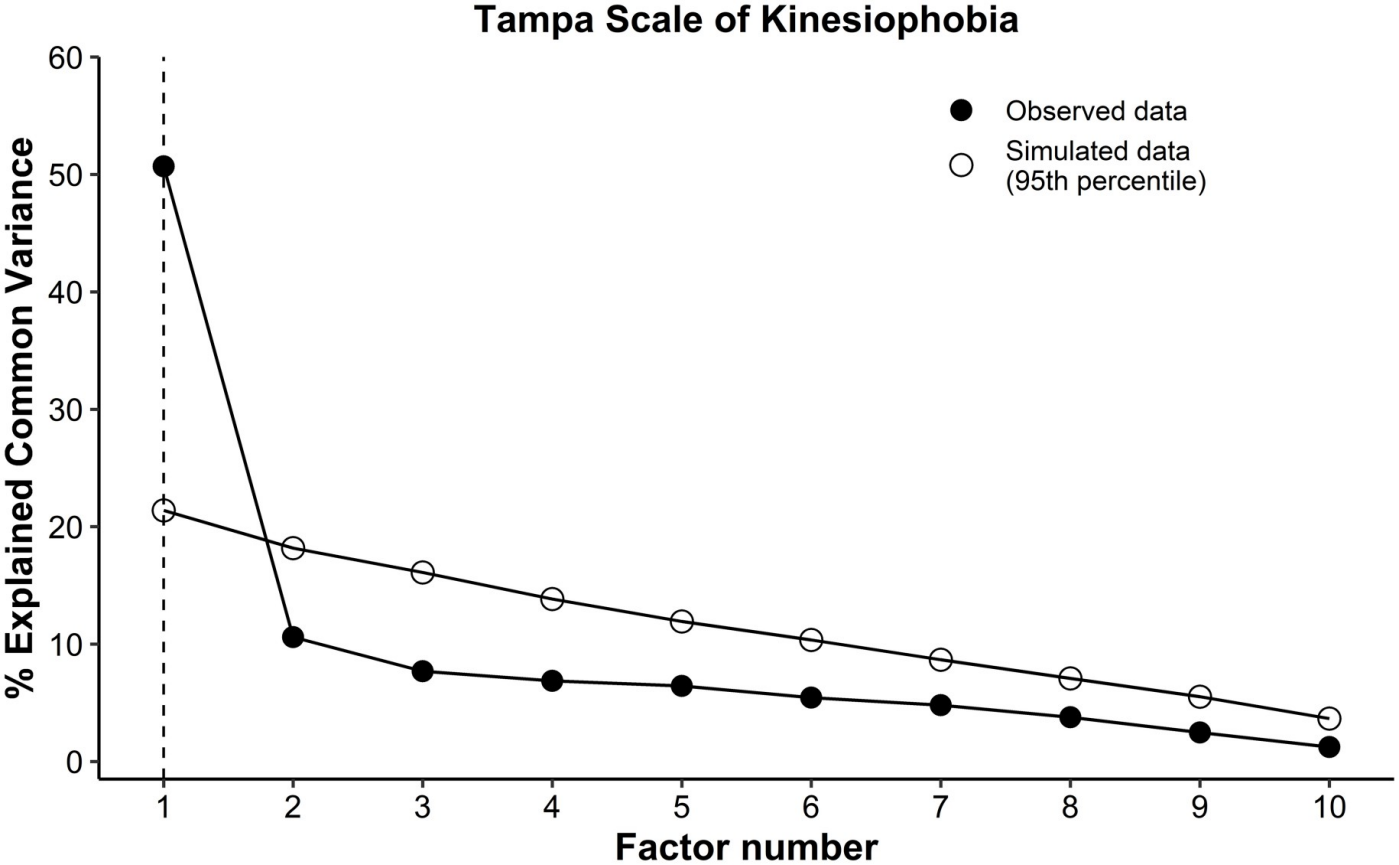
Parallel analysis of the Tampa Scale of Kinesiophobia indicated one-factor structure. The factor above the simulated data (95^th^ percentile) indicated the number of the factor in the structure.

The TSK showed statistical significance on the Bartlett’s sphericity test (p = 0.0001), and Kaiser-Meyer-Olkin test was 0.85. The exploratory factor analysis suggested 1 factor (Fig 2) that accounted for 48.34% of total variance (Table 3). The items 1, 5, 13, and 17 presented communalities lower than 0.4. Therefore, a new model (model 2) was proposed excluding those items with low value of communality and reanalyzed with an additional exploratory factor analysis, which indicated that one factor that accounted 58.49% of the variance. This new model with 7 items (items 2, 3, 6, 7, 10, 11, and 15) was considered in the analysis of the reliability, internal consistency, and responsiveness.

**Table 3:** Exploratory factor analysis of Tampa Scale of Kinesiophobia (n = 178).

Items	Factor loading	Communality
	Factor 1	
Item 1	**0.58**	0.34
Item 2	**0.69**	0.48
Item 3	**0.77**	0.59
Item 5	**0.54**	0.29
Item 6	**0.78**	0.60
Item 7	**0.76**	0.58
Item 10	**0.64**	0.41
Item 11	**0.86**	0.74
Item 13	**0.54**	0.29
Item 15	**0.64**	0.40
Item 17	**0.51**	0.26
Eigenvalue	5.31	
Variance, %	48.34	

Note: Factor loadings in bold indicate values above 0.4.

FABQ factor 1 and 2 showed moderate and good reliability, respectively. TSK with 7 items showed moderate reliability (Table 4). Internal consistency was considered adequate in all questionnaires (Table 4). FABQ factor 1 showed floor effect, with 22% of the sample achieved the lowest score. The FABQ factor 2 and the TSK did not present floor or ceiling effects.

**Table 4:** Reliability and internal consistency analysis of Fear-Avoidance Beliefs Questionnaire and Tampa Scale of Kinesiophobia (n = 86).

Variables	Mean ± SD		ICC_(3,1)_ (95% CI)	Cronbach’s Alpha	SEM	MDC_90_
	Test	Retest				
**FABQ**						
Factor 1–8 items (FABQ work)	15.01 ± 13.77	14.07 ± 14.06	0.85 (0.78–0.90)	0.92	3.93	9.11
Factor 2–2 items (FABQ Physical activity)	5.91 ± 3.36	5.40 ± 3.50	0.50 (0.32–0.64)	0.77	2.43	5.64
**TSK– 7 items**	15.07 ± 4.91	14.81 ± 4.27	0.74 (0.62–0.82)	0.81	2.10	4.88

Abbreviations: SD, Standard Deviation; ICC, Intraclass Correlation Coefficient (Two-way mixed model, consistency, single measurement); CI, Confidence Interval; SEM, Standard Error of Measurement; MDC, Minimum Detectable Change, FABQ, Fear-Avoidance Beliefs Questionnaire; TSK, Tampa Scale of Kinesiophobia.

The results of responsiveness are described in the Table 5. The FABQ factor 1 showed small ES and moderate SRM, and the factor 2 showed moderate ES and small SRM. The TSK showed moderate ES and SRM. None of the factors from FABQ and TSK showed adequate AUC.

**Table 5:** Responsiveness of Fear-Avoidance Beliefs Questionnaire and Tampa Scale of Kinesiophobia (n = 59).

Variables	Mean ± SD of change score	Effect size	SRM	AUC (95% CI)
**FABQ**				
Factor 1 (FABQ work)	5.72 ± 8.01	0.47	0.71	0.67 (0.49–0.84)
Factor 2 (FABQ physical activity)	2.00 ± 4.27	0.56	0.45	0.56 (0.35–0.76)
**TSK-7 items**	1.95 ± 2.83	0.52	0.68	0.55 (0.34–0.76)
**GROC**	5.31 ± 1.76	-	-	
**NPRS**	3.59 ± 2.46	1.49	1.45	0.82 (0.70–0.95)
**DASH**	22.52 ± 15.53	1.46	1.45	0.72 (0.51–0.94)
**EuroQol**-5D-3L	-0.10 ± .14	0.79	0.72	0.72 (0.54–0.89)

Abbreviations: AUC, area under de curve; CI, confidence interval; SD, Standard Deviation; FABQ, Fear-Avoidance Beliefs Questionnaire; TSK, Tampa Scale of Kinesiophobia; DASH, Disabilities of the Arm, Shoulder and Hand; NPRS, Numerical Pain Rating Scale.

## Discussion

Two and one factor were identified in the Brazilian versions of the FABQ and TSK, respectively. FABQ and TSK presented moderate to good reliability and adequate internal consistency. The FABQ factor 1 showed floor effect and the TSK and the FABQ factor 2 did not show floor or ceiling effect. FABQ and TSK do not seem to have adequate responsiveness for individuals with shoulder pain.

The exploratory factor analysis identified two factors in FABQ and one factor in TSK, which indicate a multidimensional and unidimensional structures of FABQ and TSK, respectively. A previous study [54] investigated the structural validity with principal component analysis of the English versions of FABQ and TSK in individuals with shoulder pain and identified four factors in both instruments. However, neither the Catell’s scree test nor the parallel analysis for identification of factors were considered in that previous study, which could have led to an overestimation of the number of factors [36,55]. Furthermore, the authors of that version [54] considered their factor analysis as preliminary due to the limited sample size (n = 80) and recommended future studies to confirm their results.

In the present study, the number of factors identified in FABQ was similar to those found in the Persian [56], Japanese [57], Nigerian [58], and English [12,25] versions of FABQ in individuals with low back pain [12,25,56–58]. The Swedish [59] and English [28] versions of TSK tested in individuals with chronic musculoskeletal pain and fibromyalgia conditions, respectively, also presented one factor, but with different items contributing for each factor. Nevertheless, the confirmatory factor analysis was not performed in this study due to the small sample size. Therefore, futures studies should investigate and compare the structure of the Brazilian FABQ and TSK to other models suggested by previous studies that investigated the FABQ and TSK in different languages and/or target populations.

The FABQ and TSK showed moderate to good reliability. The reliability analysis showed that the FABQ factors 1 showed good (ICC between 0.75 to 0.90) reliability, with floor effect, and adequate internal consistency (Cronbach’s alpha > 0.70). The FABQ factor 2 showed moderate reliability, no floor effect and adequate internal consistency. Floor or ceiling effects may indicate limited content validity and reduce the sensitivity of the instrument [35]. The observed floor effect may indicate that an adaptation of this questionnaire may be needed to assess individuals with shoulder pain. The item response theory analysis could be performed to examine item functioning characteristics, such as item difficulty and discrimination. This type of analysis contributes to shortening of scales and development of precise scoring, valid measures, and reliability estimates [60]. Past studies [54,61,62] investigated the reliability of the English [54,61] and Danish [62] versions of the FABQ in individuals with shoulder pain and presented conflicting results. Two studies [54,62] showed good reliability, and another study [61] showed an ICC of 0.43 for the physical activity subscale. Interestingly, none of those studies investigated the floor or ceiling effects, which is considered an important component of the measurement properties testing [35].

The TSK showed moderate reliability (ICC between 0.50 to 0.75) and no floor or ceiling effects and adequate internal consistency. One past study [54] observed an ICC of 0.84 for test-retest reliability and adequate internal consistency in one of four TSK factors of English version tested in individuals with shoulder pain. However, reliability of other three factors was not tested, internal consistency was adequate (Cronbach alpha higher than 0.70) in one factor, and floor and ceiling effects were not investigated [54].

The present study provided SEM and MDC values for FABQ and TSK. The SEM and MDC are considered more clinically applicable than ICC, and may assist clinicians and future studies to interpret whether the change of a score represents real change [63]. The results of this study showed that 3.93 and 2.43 points should be considered the SEM for factors 1and 2 of FABQ, respectively. TSK showed SEM of 2.10. According to the MDC calculated in this study, changes in FABQ factors 1 and 2 larger than 9.11 and 5.64 points should be considered relevant, respectively. The TSK showed MDC of 4.88 points.

The FABQ and TSK may not have appropriate responsiveness. Although the FABQ and TSK presented moderate or large ES or SRM, the AUC was not adequate, which indicate that they were not able to correctly identify between responders or non-responders from therapeutic interventions, according to the GROC. The fair responsiveness may be a consequence of limited content valid. Futures studies are needed to identify other questionnaires with better psychometric properties or adapt the FABQ and TSK for individuals with shoulder pain.

This study has some limitations. The results cannot be generalized to other versions of FABQ and TSK or to patients with different shoulder condition or other musculoskeletal disorders. The confirmatory factor analysis was not performed due to the small sample size of this study and should be performed in future investigations to verify the structure of the FABQ and TSK. The construct validity was not verified using hypothesis testing based on correlation between FABQ and TSK and other instruments that measure similar constructs. According to Ludenberg et al., (2011) [64] the pain-related fear, fear-avoidance beliefs, fear of movement, and kinesiophobia are different constructs but frequently used interchangeably. The “fear-avoidance beliefs” is the construct measured by FABQ, and another instrument that measures the same construct is the Fear-Avoidance of Pain Scale [64]. The TSK was designed to measure kinesiophobia, and according to Ludenberg et al., (2011) [64] there is no other instrument that measures the same construct [64]. The instruments Fear of Pain Questionnaire and Pain Anxiety Symptoms Scale measure the construct of pain-related fear [64], which is related to fear-avoidance beliefs and kinesiophobia. Fear-Avoidance Pain Scale, Fear of Pain Questionnaire, and Pain Anxiety Symptoms Scale could be used in the construct validity process of this study, but the psychometric properties of their Brazilian versions were not tested in individuals with shoulder pain. Therefore, future studies should investigate the validity, reliability, and responsiveness of the Brazilian versions of Fear-Avoidance of Pain Scale, Fear of Pain Questionnaire, and Pain Anxiety Symptoms Scale in individuals with shoulder pain.

## Conclusion

The results of this study indicate that FABQ and TSK are multidimensional and unidimensional instruments, respectively. Those instruments presented moderate to good reliability, adequate internal consistency, and the responsiveness was considered to be suboptimal in individuals with shoulder pain.

## Supporting information

S1 File (PDF)
