# Supplementary material for: Measurement properties of the Brazilian versions of Fear-Avoidance Beliefs Questionnaire and Tampa Scale of Kinesiophobia in individuals with shoulder pain
Source: PLoS One. 2021 Dec 1;16(12):e0260452. doi: 10.1371/journal.pone.0260452 (PMC8635377; doi:10.1371/journal.pone.0260452)

Supplemental file 1. Floor and Ceiling effect

Figure 1. Distribution of the FABQ factor 1.

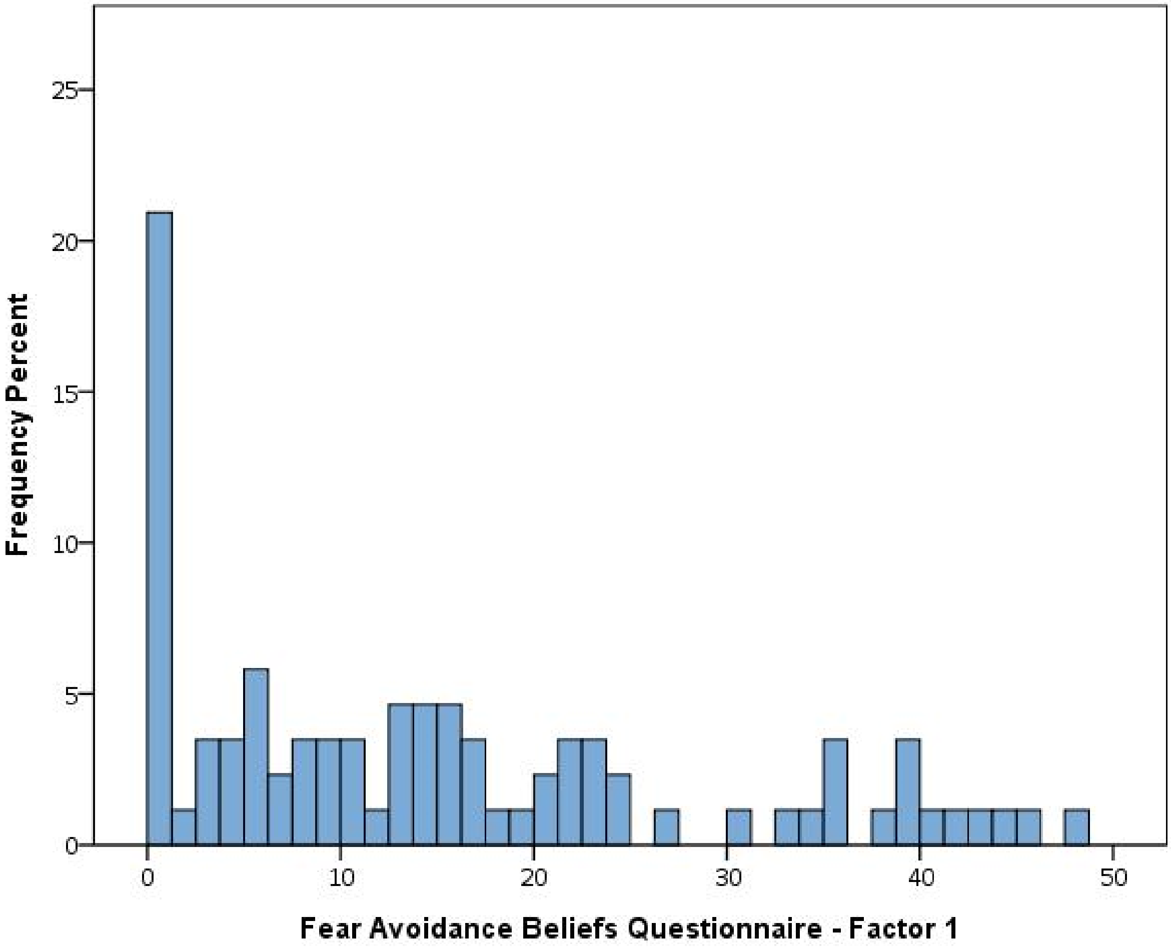

Figure 2. Distribution of the FABQ factor 2.

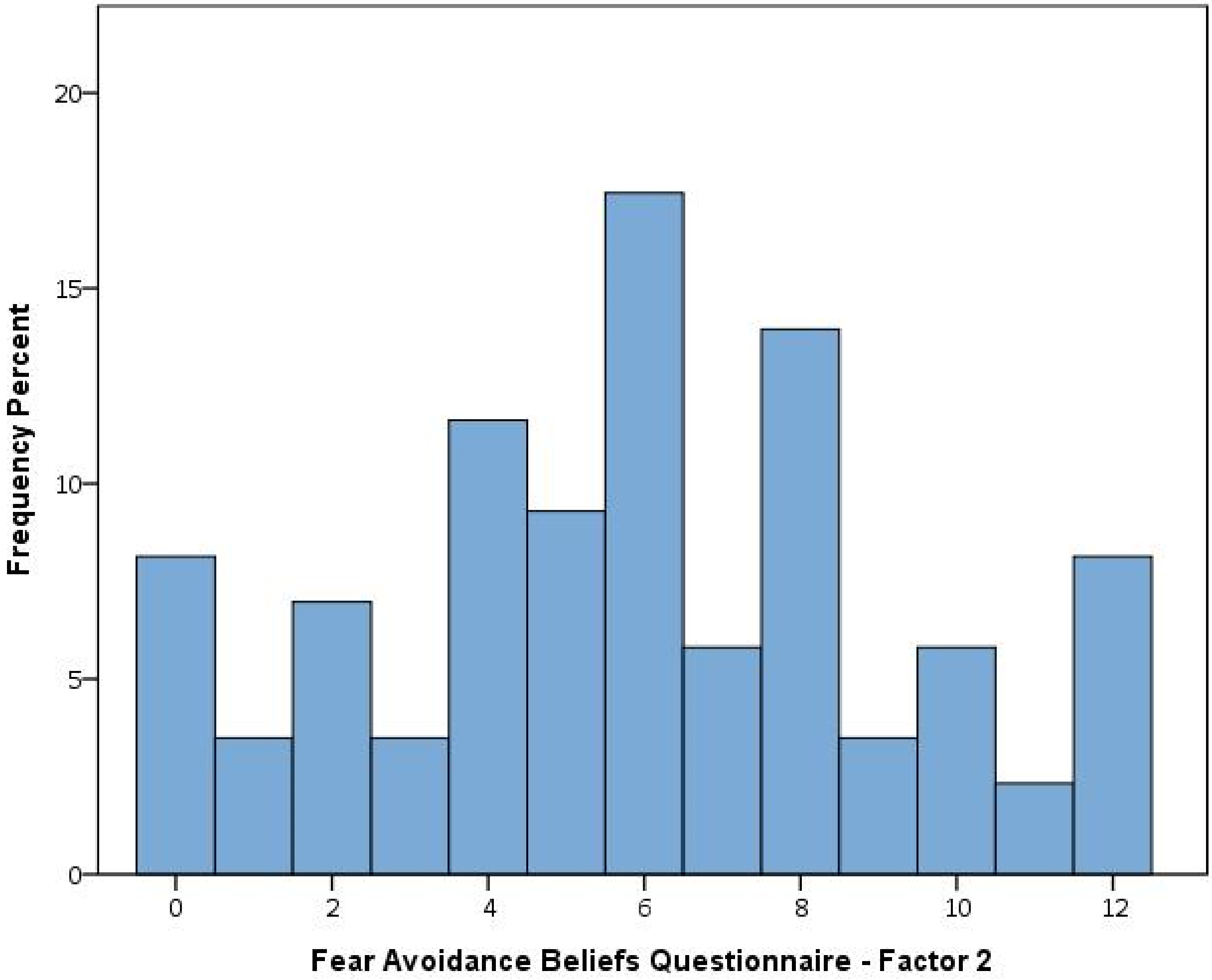

Figure 3. Distribution of the TSK.

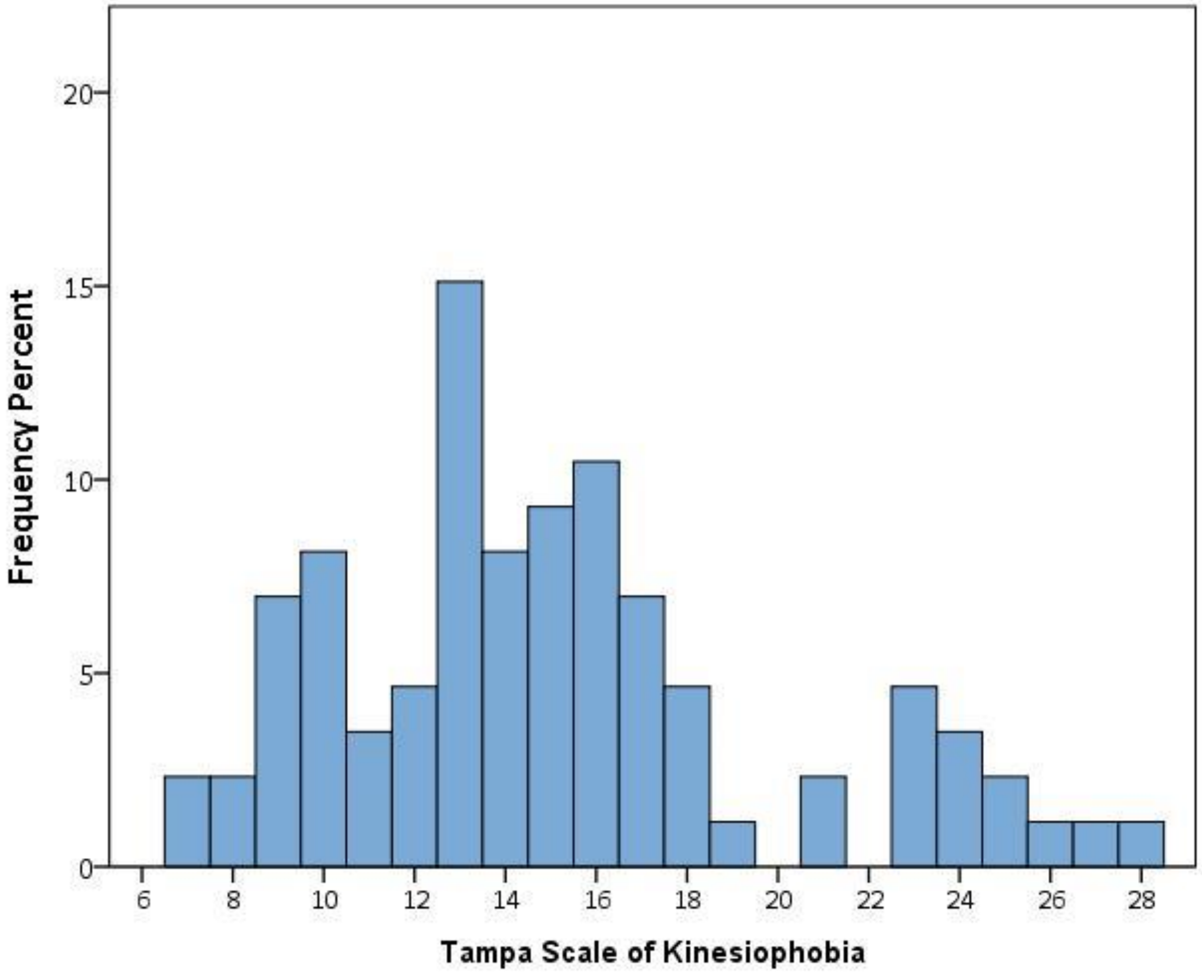

Supplement: S1 File — (PDF) [file pone.0260452.s001.pdf]
